# Ectopic Supernumerary Fourth Molar in the Mandibular Notch: A Report of an Atypical Case With Literature Review

**DOI:** 10.7759/cureus.89557

**Published:** 2025-08-07

**Authors:** Sara Macho-Lopez, Jose-Francisco Montes-Carmona, Alberto Garcia-Perla-Garcia, Rafael Martinez-De-Fuentes, Pedro Infante-Cossio

**Affiliations:** 1 Department of Oral and Maxillofacial Surgery, Virgen del Rocio University Hospital, School of Medicine, University of Seville, Seville, ESP; 2 Department of Prosthodontics, School of Dentistry, University of Seville, Seville, ESP

**Keywords:** dentigerous cyst, ectopic tooth, fourth molars, mandibular notch, sigmoid notch, supernumerary teeth

## Abstract

The presence of a fourth supernumerary molar in the mandible represents a rare anomaly of dentofacial development. Here, we describe an exceptional, previously unreported case of a 33-year-old man with a solitary ectopic supernumerary fourth molar located in the sigmoid notch, which was associated with a dentigerous cyst. In addition, a review of the literature related to this uncommon and atypical clinical presentation is provided. The patient presented with persistent pain and intermittent swelling on the ipsilateral side of his face. Previous radiographs revealed that, over a 10-year period, the fourth molar had migrated from a position distal to the third molar to the mandibular notch. Surgical extraction of the supernumerary tooth and enucleation of the associated cyst via an intraoral approach averted the cyst recurrence and other complications. This clinical report highlights the importance of early detection and surgical intervention to prevent cyst formation and the atypical migration of supernumerary teeth. It also emphasises the need for periodic follow-up to avoid associated complications.

## Introduction

A supernumerary fourth molar, also known as a distomolar, is a rare developmental dentofacial anomaly characterized by the presence of an additional molar distal to the third molar [1]. It is usually solitary and small, predominantly occurring in the maxilla (92%), and typically remaining unerupted (82.8%) [2]. A global prevalence of 0.32% has been reported [2].

The presence of a supernumerary fourth molar in the mandible can pose diagnostic and therapeutic challenges due to the possible absence of initial symptoms and the anatomical complexity of the region [3]. It is usually asymptomatic in its early stages and is often discovered incidentally during a routine radiograph. However, over time, it may present clinically with symptoms such as swelling, pain, trismus, or recurrent infections. Potential complications include impaction, displacement or root resorption of adjacent teeth, dental malposition, development of cystic lesions or odontogenic tumours, and ectopic or delayed eruption [4]. In rare cases, these supernumerary teeth can be found in ectopic locations, such as the mandibular ramus or the coronoid process [5-10]. This study reports the first case of an ectopic supernumerary fourth molar (ESFM) located in the sigmoid notch and associated with a dentigerous cyst in a non-syndromic patient. We also review the literature on this rare and atypical clinical presentation.

## Case presentation

**Figure 1:**
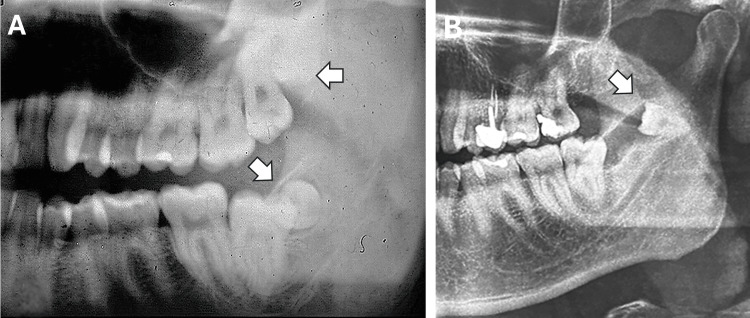
Conventional panoramic radiograph views. (A) Previous panoramic radiograph taken 10 years earlier showing two impacted rudimentary fourth molars (upper and lower) on the left side (arrows). (B) Current panoramic radiograph showing the ectopically positioned, eumorphic mandibular fourth molar in the sigmoid notch (arrow). An ascending radiolucent image was seen extending from the impacted third molar to the sigmoid notch.

 A 33-year-old man came to our clinic complaining of a dull, persistent pain and intermittent swelling on the left side of his face, which had lasted for three months. At the age of 23, he was diagnosed with two impacted left supernumerary fourth molars (upper and lower), which had a rudimentary appearance (Figure 1A). His dental history revealed that he had undergone maxillary fourth molar extraction under local anaesthesia 10 years earlier. The extraction of the mandibular fourth molar was scheduled, but the patient refused treatment and did not return for a follow-up appointment. The patient reported no other significant medical history.

**Figure 2:**
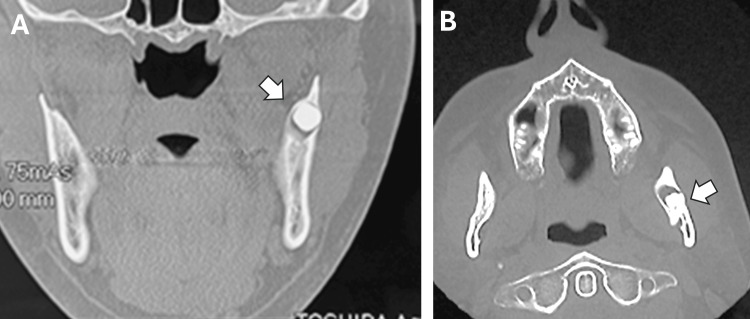
Computed tomography with coronal, sagittal, and axial slices. (A) Coronal and (B) axial CT scans showing a well-defined radiolucency around the crown, suggesting the formation of a dentigerous cyst (arrows).

The current panoramic radiograph revealed a molar-type supernumerary tooth located ectopically in the sigmoid notch region (Figure 1B). In addition, an ascending radiolucent image was observed, showing a trajectory from the impacted third molar to the sigmoid notch along the mandibular ramus. Comparing this with a radiograph taken 10 years earlier revealed that the ESFM had migrated atypically along the mandibular ramus over the years and that changes had occurred in the morphology of the rudimentary supernumerary until its crown and roots were fully formed. Computed tomography (CT) revealed well-defined, unilocular radiolucency around the crown of the supernumerary molar, consistent with a dentigerous cyst (Figure 2).

**Figure 3:**
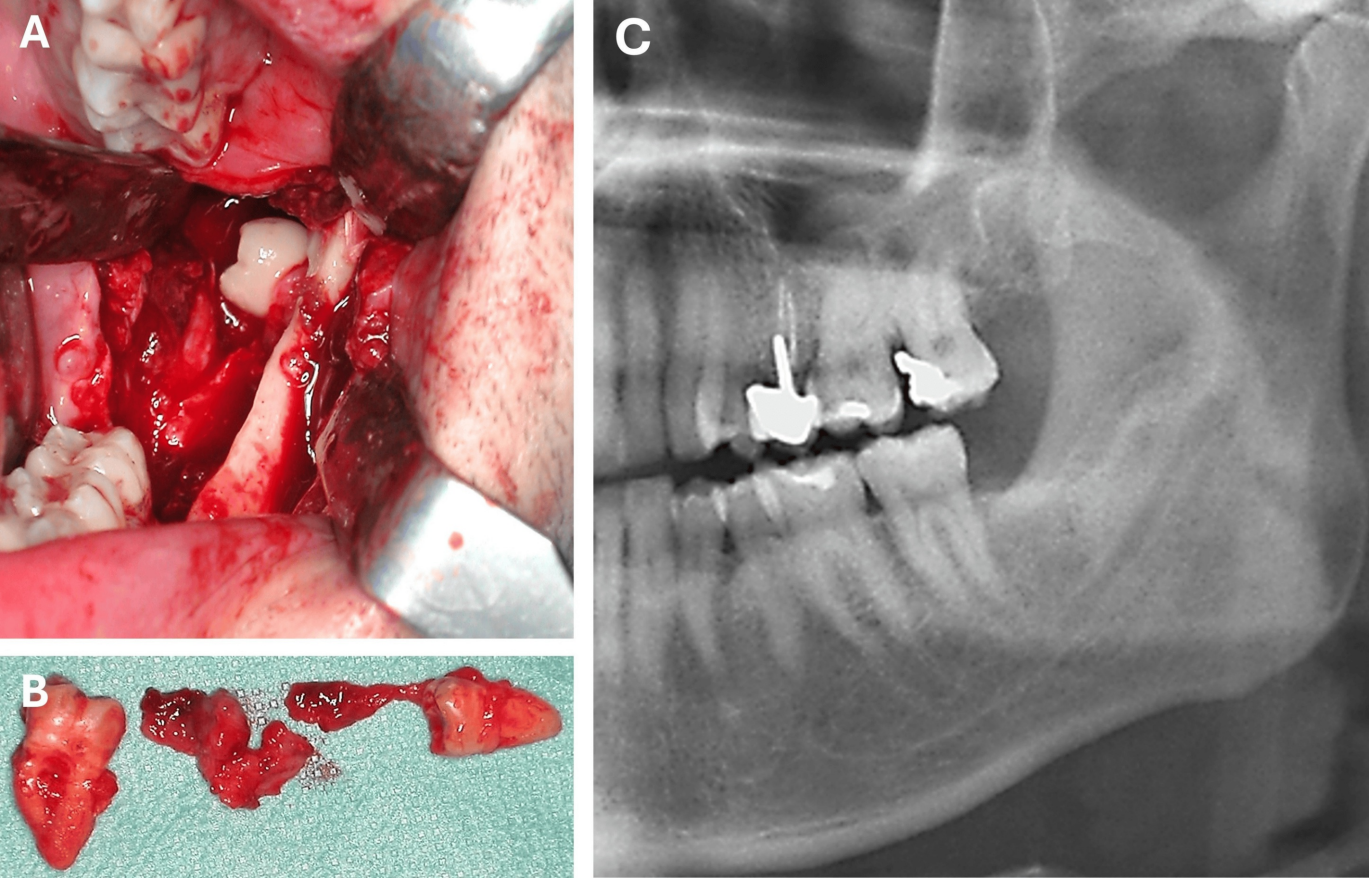
Intra-operative images and post-operative panoramic radiograph. (A) Intraoral approach to the impacted fourth molars and an adjacent dentigerous cyst. (B) Extraction of the left third and fourth molars, together with enucleation of the associated cyst. (C) Five-year follow-up panoramic radiograph.

The patient was admitted for the surgical extraction of the lower third and fourth molars and the enucleation of the associated cyst. The procedure was performed intraorally using a drill under general anaesthesia (Figures 3A, 3B). Histological examination revealed tissue consistent with a dentigerous cyst around the ESFM. The postoperative course was uneventful. Subsequent follow-up examinations showed a favourable outcome with complete resolution of swelling and pain and no recurrence of the cyst. A follow-up panoramic radiograph taken five years later showed evidence of bone regeneration (Figure 3C).

## Discussion

The finding of an ESFM located in the sigmoid notch region and associated with a dentigerous cyst is an exceptional occurrence that, to the authors' knowledge, has not been previously reported. The overall prevalence of supernumerary fourth molars in the mandible is estimated to be approximately 0.02% [2], yet there are few studies on an ESFM in this location [5-10]. Therefore, the true frequency of this anomaly is unknown and is likely to be higher than reported due to undocumented cases.

The causes contributing to the formation of supernumerary fourth molars remain unclear. Alterations in odontogenesis, such as hyperactivity and abnormal proliferation of the dental lamina, as well as splitting of the tooth bud, have been hypothesised [5,9]. Certain genetic factors associated with craniofacial syndromes have also been identified [4]. Due to their rarity, much of the available information has been extrapolated from studies on ectopic third molars, which have revealed the relationship between these molars and dentigerous cysts [11]. Dentigerous cysts originate from the accumulation of fluid between the reduced enamel epithelium and the crown of an impacted supernumerary tooth [6].

A review of the English literature [6-10], including the presented case, revealed a total of six cases of an ESFM in the mandibular ramus associated with dentigerous cysts (Table 1). Analysis of these cases showed that the mean age at diagnosis was 48.3 years, with a higher prevalence in women (66.7%). Most cases were located in the ascending mandibular ramus, with one case in the coronoid region and one in the sigmoid notch. The predominant signs and symptoms observed included ipsilateral pain, swelling, and limited mouth opening. Surgical extraction was chosen in all cases, mainly via an intraoral approach (66.7%). Postoperative complications were rare, with haematomas and long-lasting scarring being the most common.

**Table 1:** Reported cases of ectopic mandibular fourth molars associated with dentigerous cysts in the mandibular ramus.

Authors/year	Age/Gender	Fourth molar location	Signs and symptoms	Treatment/complications
McCrea 2009 [6]	60/Female	Mid-ramus region	Swelling	Intraoral surgical removal/Hematoma
Sanghera and Jones 2013 [7]	49/Female	Mid-ramus region	Swelling, pain, limited mouth opening, and parotid fistula	Extraoral surgical removal (preauricular approach)/ Scarring and long-standing facial sinus
Mathew et al. 2017 [8]	46/Male	Coronoid region	Swelling, pain, and limited mouth opening	Intraoral surgical removal/No
Singleton and Nastri 2018 [9]	59/Female	Mid-ramus region	Swelling, pain, and limited mouth opening	Extraoral surgical removal (transparotid approach)/ None
Hara et al. 2019 [10]	43/Female	Mid-ramus region	Pain and limited mouth opening	Intraoral surgical removal/None
Presented case 2025	33/ Male	Sigmoid notch region	Swelling and pain	Intraoral surgical removal /None

These cases suggest that, while most patients are initially asymptomatic, they eventually exhibit symptoms due to compression of adjacent structures or the development of associated lesions. This causes recurrent pain, inflammation and limited mouth opening [8-10]. The presented clinical case demonstrates that complications such as cyst formation or ectopic migration could have been avoided through early diagnosis and timely treatment with regular follow-ups. A comparison of the panoramic radiograph with one taken 10 years earlier revealed that the ESFM had undergone unusual migration along the mandibular ramus over time. The current panoramic radiograph revealed a radiolucent path extending from the third molar to the sigmoid notch, potentially indicating the route taken by the ESFM during migration. The progressive transformation of the tooth's shape was also evident, culminating in the formation of a complete crown and root. These radiographic findings suggest that cystic pressure and progressive morphological changes to the supernumerary tooth may have contributed to its ectopic migration along the ramus. Furthermore, an abnormal eruption pattern or malposition of tooth germs may also have contributed to ectopia [9]. In our clinical case, although the migration of the supernumerary molar observed during the 10-year radiographic follow-up is documented on imaging, the interpretation that this displacement was caused by a dentigerous cyst should be considered with caution due to the complexity of the factors that can influence dental ectopia and the inherent difficulty of establishing a direct causal relationship based solely on radiological evidence. Although the radiographic images allowed us to observe the change in the position of the ESFM and the concomitant presence of a cystic lesion, this association should be interpreted as a plausible hypothesis.

Radiological studies play a fundamental role in the diagnosis, localization, identification, and treatment planning of supernumerary molars [12]. Panoramic radiography is routinely used as the first imaging tool, as it allows visualization of the presence, number, and position of ectopic supernumerary fourth molars. In addition, three-dimensional tomography, such as cone beam computed tomography or conventional helical CT, offers greater accuracy in determining the exact location of the molar in relation to anatomical structures as well as detecting possible associated abnormalities, such as dentigerous cysts or other bone lesions. In our clinical cases, a CT scan was essential for assessing the relationship between the ESFM and the inferior dental canal and cortical bone. It was also necessary to identify the cyst and distinguish it from aggressive lesions, such as ameloblastoma.

Unerupted supernumerary teeth are frequently associated with dentigerous cysts, primarily due to the prolonged persistence of the dental follicle around the crown of the impacted tooth. In the clinical case presented, a well-demarcated unilocular radiolucent image was detected associated with the crown of the ectopic supernumerary tooth, consistent with a dentigerous cyst. Based on the patient's history and the clinical and radiographic findings obtained by panoramic radiography and CT, our presumptive diagnosis was clear, and therefore, standard treatment was proposed, which consisted of complete excision of the cyst along with the associated impacted tooth and examination of the surgical specimen by the Pathology Department for definitive histopathological confirmation.

The ESFM requires an individualised approach. The surgical treatment varies depending on the location and position of the ectopia, its proximity to critical structures and the patient's comorbidities. Extraction is clearly indicated in cases involving complications such as ectopic eruption, persistent pain, swelling, TMJ dysfunction, or the development of associated pathologies including cysts, tumours and infections. Timely intervention can prevent serious complications, such as cyst formation and atypical tooth migration. The standard treatment consists of extraction of the supernumerary tooth together with cystectomy, via an intraoral or extraoral approach. Intraoral approaches are preferable in order to avoid visible scarring and damage to the facial nerve. However, extraoral approaches may be required in complex cases involving a very distant ectopic position. The most common access routes are the preauricular, transmasseteric, anteparotid, submandibular, and retromandibular [7,9]. Endoscopy is a less invasive treatment option resulting in lower operative morbidity [13].

In the absence of immediate symptoms or risks, a preventive extraction or a wait-and-see approach may be preferred, leaving the supernumerary tooth in situ until surgical treatment is performed. While the decision to remove an ESFM ultimately depends on the patient, if a conservative approach is chosen, close observation with regular radiographic monitoring is recommended. Furthermore, long-term follow-up at frequent intervals is essential, even after treatment, to prevent cyst recurrence and other complications. However, it should be acknowledged that the available evidence on ESFM in the mandible is based on a very small number of published clinical cases, which considerably limits the ability to generalize conclusions about all potential risks and define management guidelines. Therefore, we emphasize the need for further research and studies with larger sample sizes to support future evidence-based management protocols.

## Conclusions

In this paper, we present the first clinical case of an ESFM located in the sigmoid notch and associated with a dentigerous cyst, occurring in a non-syndromic patient. We emphasise the importance of early detection and surgical intervention to avert cyst formation and the atypical migration of supernumerary teeth. Periodic follow-up is also essential to prevent associated complications. An individualized treatment plan is necessary, considering the location and position of the ectopia, as well as the risks and benefits of surgical removal.
